# Advantages of a Structured Conditioning Program to Optimize the Aerobic Capacity and Functional Independence of a Patient With Acute Inflammatory Demyelinating Polyneuropathy

**DOI:** 10.7759/cureus.31647

**Published:** 2022-11-18

**Authors:** Aditi Nagore, Snehal Samal, Rakesh K Kovela, Vikrant G Salphale

**Affiliations:** 1 Department of Physical Therapy, Datta Meghe Institute of Medical Sciences, Wardha, IND; 2 Department of Neuro Physiotherapy, Ravi Nair Physiotherapy College, Datta Meghe Institute of Medical Sciences, Wardha, IND; 3 Department of Physiotherapy, Nitte Institute of Physiotherapy, NITTE (Deemed to be University), Mangalore, IND

**Keywords:** guillain-barre syndrome, neuro-rehabilitation, pain management, neurophysiotherapy rehabilitation, acute demyelinating inflammatory polyneuropathy

## Abstract

Acute demyelinating inflammatory polyneuropathy is a variant of Guillain-Barre syndrome (GBS) - an asymmetrical condition that primarily affects the segment of the peripheral nervous system. Weakness or tingling sensations that be commenced in the inferior limbs and progress to the brachium and face are early signs of this condition. Physiotherapy plays a very crucial role in acute demyelinating inflammatory polyneuropathy in combination with medical management. Criteria through which the clinicians conclude the same are marked affection in the proximal musculature, lower motor neuron type of manifestation, and commencement of symptoms in an ascending sequence. In this study, we presented a case of a 62-year-old male who manifested with the complaint of bilateral superior and inferior limb weakness and was admitted to our hospital. He was diagnosed with acute demyelinating inflammatory polyneuropathy after investigations, such as a lumbar puncture, which revealed a raised level of proteins in cerebrospinal fluid (CSF). With these complaints, he was referred to the physiotherapy wing, and physiotherapy rehabilitation was commenced. Thus, we concluded from this study that in the case of acute demyelinating inflammatory polyneuropathy, physiotherapy rehabilitation was proven to be fruitful in the speedy recovery of the patient and preventing secondary complications along with improving strength and activities of daily living (ADLs) and enhancing the overall quality of life.

## Introduction

According to the World Health Organization, acute demyelinating inflammatory polyneuropathy is asymmetrical, primarily motor neuropathy affecting multiple peripheral nerves occurs as a result of hypersensitivity to unknown viruses or allergens. Acute demyelinating inflammatory polyneuropathy is the most prevalent cause of neuromuscular paralysis in the Western world [1]. It affects both sexes at any age, peaking between the ages of 20 and 50; males are affected more than females, and it can occur at any time of year, with an incidence of 1 to 2 per 100,000 people [2]. In acute demyelinating inflammatory polyneuropathy, the body’s immune system attacks the segment of the peripheral nervous system. The etiology of acute demyelinating inflammatory polyneuropathy is unknown. The patient with acute demyelinating inflammatory polyneuropathy could be affected by a bacterial or viral infection. And it can also be caused by vaccinations or surgery. Its early signs include weakness or tingling sensations that commence in the lower body [3] and progress to the arms and face [4]. The symptoms can appear anywhere from a few hours to a few weeks. It is a life-threatening situation that requires the patient to be treated in an intensive care unit. The investigation to detect acute demyelinating inflammatory polyneuropathy is the cerebrospinal fluid (CSF) examination, which detects the increased albumin level in the CSF, clinical history and symptoms supported by lumbar puncture, and electromyography. Acute demyelinating inflammatory polyneuropathy is managed by both medical management and physiotherapy management. Medical management includes plasmapheresis, steroid therapy, and injection of IgG. Physiotherapy management executes a major role in the treatment and early recovery of the acute demyelinating inflammatory polyneuropathy patient, which includes preserving a range of motions at all joints, withstanding muscle properties, prevention and treatment of pressure sores, functional training, and gait training. Thus, it optimizes the patient’s functional potency and endurance and gives psychological support.

## Case presentation

Patient information

A 62-year-old male visited the Neuro Physiotherapy Department with a complaint of bilateral weakness in the lower limb for 10 days. The patient was apparently alright 10 days back; then he was complaining of weakness in the dual lower limb. After two days, the patient was unable to walk; therefore, he was taken to the nearby hospital. However, at the same time, he experienced weakness in both upper limbs. When an examination was done, it revealed raised blood pressure, and he was advised to take medications for the same. The next day, he felt marked weakness in the lower and upper limbs, which aggravated day by day. So, with this complaint, he approached the rural hospital for further management and was admitted into the medical ward. He underwent a CSF examination, which revealed an increment in the level of proteins in the CSF, and a diagnosis of acute demyelinating inflammatory polyneuropathy was made. The patient was referred to physiotherapy for the same complaint, and physiotherapy rehabilitation was started. During rehabilitation, the patient was evaluated in a bedside sitting position his vitals were stable and his body build type was endomorphic. The patient was having a kyphotic posture. There was a marked amount of tightness in the muscles around the hip joint due to prolonged immobilization. As soon as the first symptom developed, the physiotherapy session commenced after two weeks. The tone in the inferior extremities was normal. Superficial reflexes were altered, + on reflex grading and deep reflexes were intact. His balance was hampered due to the physiological inefficiency of the muscles. No respiratory weakness or respiratory support was given to the patient as his respiratory care was good.

Diagnostic evaluation

The complete blood count, kidney function test, liver function test, and lipid profile examination were carried out, but there was no alteration in any of these examinations. A CSF examination was done, which revealed high levels of protein, as a result of albuminocytological dissociation.

Pharmacological treatment

The patient was managed by a parenteral administration of IgG once a day for three days.

Therapeutic Intervention

The rehabilitation protocol is shown in Table 1.

**Table 1:** Therapeutic intervention.

Phase 1 (0-7 days)	Phase 2 (2-4 weeks)	Phase 3 (4-8 weeks)	Phase 4 (Home Exercise Program)
Pursed lip breathing progressed later to diaphragmatic breathing to overcome the breathlessness with repetitions 10 times, thrice a day. Ankle pumps 15-20 times to prevent vascular consequences. Static exercises 10 times twice a day to enhance the stability of joints. The patient had received passive movements of the right lower limb, as shown in Figure 1.	Dynamic exercises blended with an active assisted range of motion activities for superior and inferior limbs 10 times, twice a day. Stretching of biarticular hamstring and quadriceps muscles thrice a day, 3 repetitions, with a hold of 5-10 seconds. Pelvic bridging for 10 times with a hold of 5 seconds and thrice a day. The patient had received passive heel slides, as shown in Figure 1.	Active free exercises for the joints of superior and inferior limbs followed by resistance training through weight cuffs of the optimal load 10 times, thrice a day. Sit-to-stand training followed by standing with the help of a walker followed by walking with the help of a walker, dual crutches through a four-point gait, and later on with a two-point gait for 6 minutes, thrice a day. In the end, walking with a cane was mastered.	Repetition of exercises in phases 2 and 3 and be cautious about the complications. The patient was told to revisit the hospital after 15 days for a follow-up.

**Figure 1:**
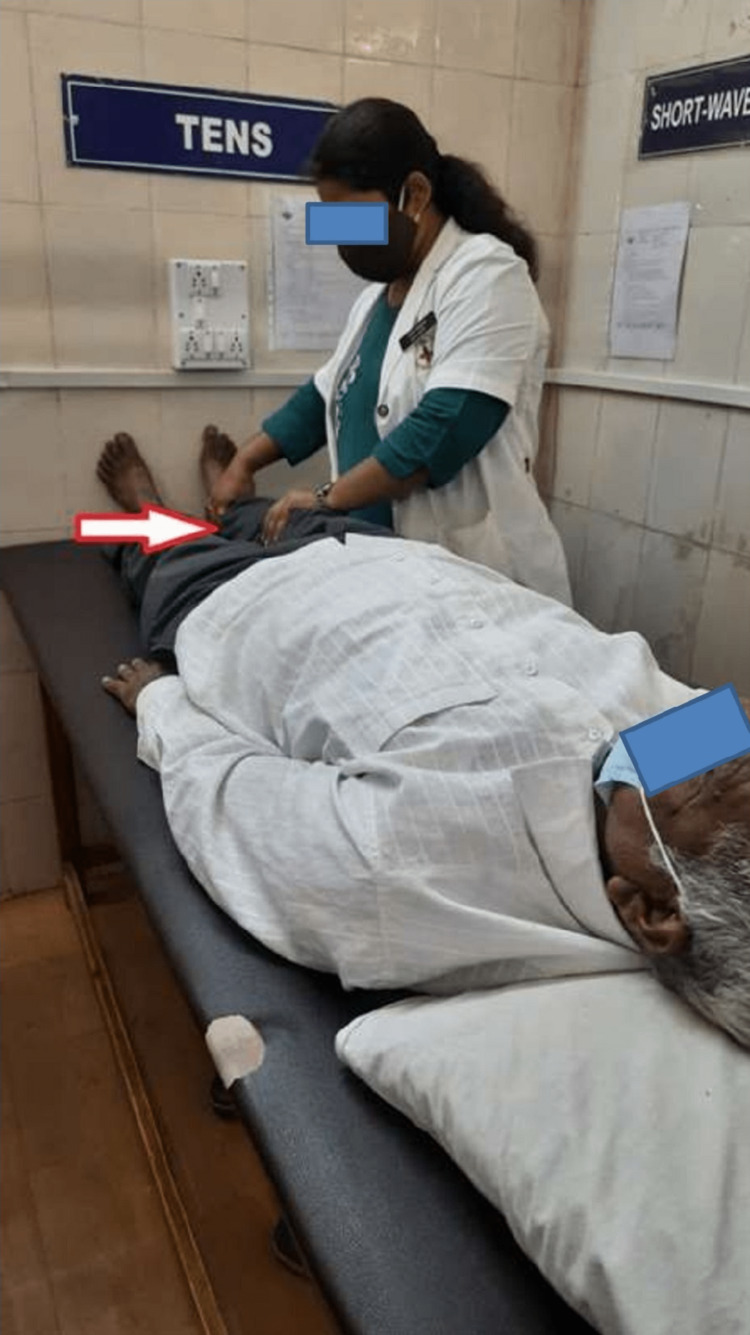
Passive movement of the right lower limb by the therapist.

**Figure 2:**
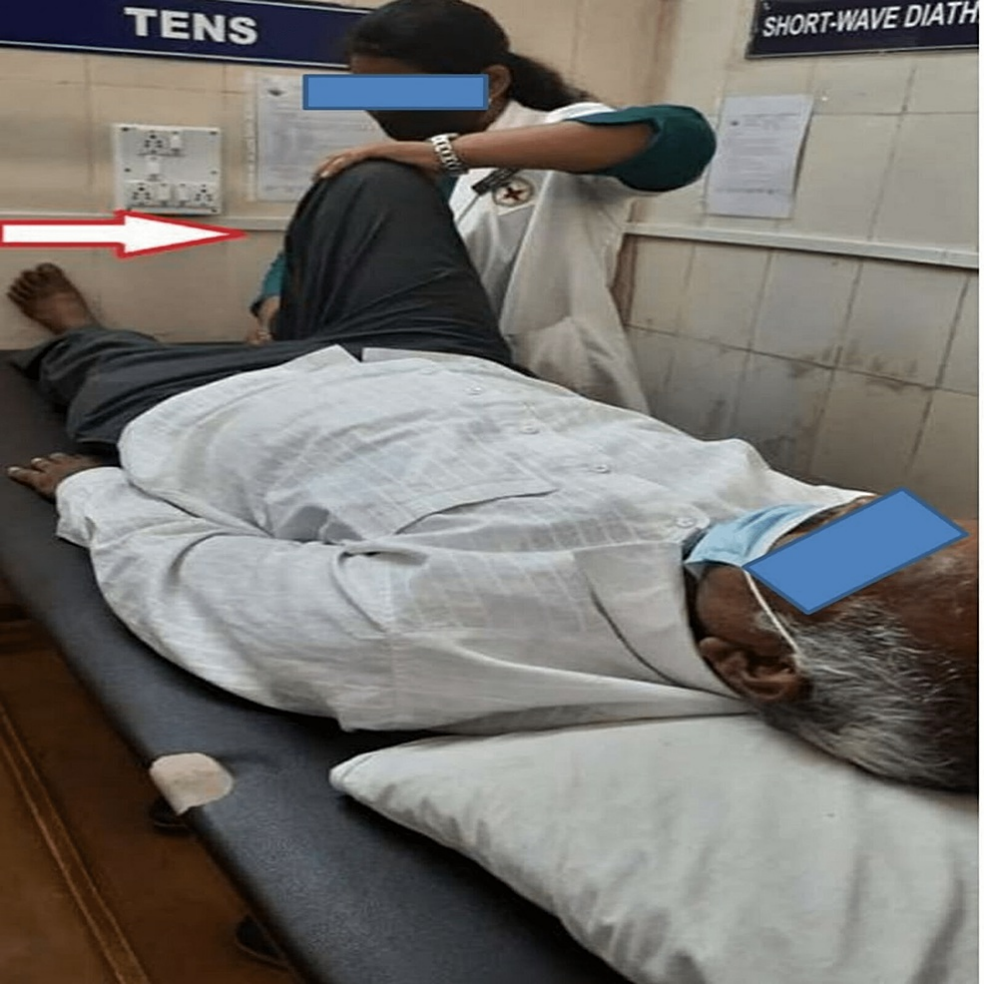
Passive heel slides being performed by the therapist.

Passive movement of the right lower limb is shown in Figure 1, and passive heel slides are shown in Figure 2.

Follow-up and outcome

After 21 days of treatment, the patient came to the physiotherapy venture for reevaluation. Following the examination, the patient was taught advanced exercises. His treatment plan included 30-minute sessions three times a week. The following measurements are shown in Table 2.

**Table 2:** Follow-up and outcome measures.

Outcome measures	Pretreatment	Posttreatment
Berg balance scale	20/56	35/56
Functional independent scale	96/126	108/126

Manual muscle testing for bilateral upper and lower limbs is shown in Tables 3 and 4.

**Table 3:** Manual muscle testing.

Upper limb joints	Right	Left
Shoulder - flexion	3/5	3/5
Extension	3/5	3/5
Adduction	3/5	3/5
Abduction	3/5	3/5
Lateral rotation	3/5	3/5
Medial rotation	3/5	3/5
Elbow - flexion	3/5	3/5
Extension	3/5	3/5
Wrist - flexion	3/5	3/5
Extension	3/5	3/5
Lower limb joints		
Hip - flexion	3/5	3/5
Extension	3/5	3/5
Adduction	3/5	3/5
Abduction	3/5	3/5
Knee - flexion	3/5	3/5
Extension	3/5	3/5
Ankle - dorsiflexion	3/5	3/5
Plantar flexion	3/5	3/5

**Table 4:** Range of motions.

Joints	Right	Left
Shoulder - flexion	0°-92°	0°-97°
Extension	0°-20°	0°-24°
Abduction	0°-83°	0°-79°
Lateral rotation	0°-60°	0°-62°
Medial rotation	0°-50°	0°-57°
Elbow - flexion	0°-140°	0°-140°
Wrist - flexion	0°-80°	0°-83°
Extension	0°-73°	0°-75°
Hip - flexion	0°-80°	0°-83°
Extension	0°-10°	0°-9°
Adduction	0°-10°	0°-13°
Abduction	0°-30°	0°-27°
Knee - flexion	0°-86°	0°-95°
Extension	86-0°	95-0°
Ankle - dorsiflexion	0°-18°	0°-20°
Plantar flexion	0°-38°	0°-40°

## Discussion

Acute demyelinating inflammatory polyneuropathy is asymmetrical, primarily motor neuropathy affecting multiple peripheral nerves as a result of hypersensitivity to unknown viruses or allergens. Weakness or tingling sensations commence in the lower body and progress to the brachium and face - immediate signs of acute inflammatory demyelinating polyneuropathy [5]. In the treatment of acute inflammatory demyelinating polyneuropathy, neuromuscular electrical stimulation plays a vital role in achieving faster recovery [6]. However, it has been reported that between 8% and 16% of acute demyelinating inflammatory polyneuropathy patients experience one or more deteriorations after initial improvement or stabilization following plasmapheresis or intravenous Ig (IVIg) treatment. Pitetti et al. conducted a study on rehabilitation outcomes of patients who developed acute demyelinating inflammatory polyneuropathy that seems to be in line with this study [7]. Rasch scores functional independence measure (FIM) as well as acute and rehabilitation charges in Guillain-Barre syndrome (GBS). The patient's functional capacity increased, which improved his quality of life and functional outcome. This study seems to be in line with previous research conducted by Karavatas [8]. They illustrated the role of neurodevelopmental sequencing in the physical therapy management of a geriatric patient with GBS. In this case, the patient received a structured physiotherapy regime for conditioning, and as per the result, we came to know that it improved the patient’s functional capacity and endurance and gave psychological support to the patient. Thus, this case study suggests that physiotherapy has a substantial impact on improving strength and activities of daily living (ADLs) and enhancing the overall quality of life.

## Conclusions

We concluded that a structurally organized conditioning program assists in achieving optimal aerobic capacity as well as functional independency in patients affected by acute inflammatory demyelinating polyneuropathy. Physical therapy rehabilitation has been shown to enhance patients' ADLs and quality of life and hence a quick recovery. Despite not fully recovering after rehabilitation, the patient's range of motion and muscle strength were improved.
